# OP24 How Does Robotic-Assisted Surgery Real-World Evidence Complement Randomized Controlled Trials Evidence? A Systematic Literature Review And Meta-Analysis

**DOI:** 10.1017/S0266462324000874

**Published:** 2025-01-07

**Authors:** Neera Patel, Ana Yankovsky, April Hebert, Usha Kreaden

## Abstract

**Introduction:**

The use of real-world evidence (RWE) by health technology assessments to evaluate emerging technologies has increased. Although traditionally recognized as the gold standard of evidence, randomized controlled trials (RCTs) may be challenging to conduct, especially in surgical settings. The aim of this analysis is to synthesize and compare results from RWE with those from RCTs for robotic-assisted surgery (RAS).

**Methods:**

A systematic review was performed according to PRISMA methods. RWE and RCT studies published between 1 January 2010 and 31 December 2022 and comparing RAS, laparoscopic, or open surgery across seven procedures were included. Perioperative outcomes of interest were operative time, length of stay (LOS), conversion to an open procedure, estimated blood loss (EBL), blood transfusions, readmissions, reoperations, postoperative complications, and mortality. A meta-analysis was performed using R software.

**Results:**

Thirty-three RCTs and 121 RWE studies were included. For RAS versus laparoscopy, RCTs and RWE were concordant for conversions (RCT:OR=0.56 [0.42, 0.74], p<0.01; RWE:OR = 0.41 [0.36, 0.47], p<0.01) and LOS in favor of RAS (RCT:WMD = −0.66 [−1.12, −0.20], p<0.01; RWE:WMD = −0.50 [−0.63, −0.36], p<0.01), while operative time was longer for RAS (RCT:WMD = 27.89 [12.66, 43.12], p<0.01; RWE:WMD = 28.89 [15.56, 42.22], p<0.01). RWE complemented RCTs on blood transfusions and mortality, showing RAS favored over laparoscopy. For RAS versus open surgery, RCTs and RWE agreed RAS had significantly lower EBL (RCT:WMD = −260.42 [−515.16, −5.67], p = 0.05; RWE: WMD = −328.27 [−474.08, −182.47], p<0.01), lower postoperative complications (RCT:OR = 0.70 [0.50, 0.97], p = 0.03; RWE:OR = 0.56 [0.50, 0.62], p<0.01), shorter LOS (RCT:WMD = −1.88 [−3.12, −0.64], p<0.01; RWE:WMD = −1.95 [−2.20, −1.70], p<0.01), and longer operative time (RCT:WMD = 35.38 [2.14, 68.61], p=0.04; RWE:WMD = 38.80 [24.62, 52.97], p<0.01). For the remaining outcomes, RCTs showed no difference, while RWE provided complementary results in favor of RAS.

**Conclusions:**

RWE confirmed many of the results shown in RCTs and complemented findings for perioperative outcomes. Based on these results, RWE can supplement the findings from RCTs in the literature, provide more generalizability, and offer a more comprehensive landscape of the evidence on robotic-assisted surgery.

